# Wind-Induced Pressure Prediction on Tall Buildings Using Generative Adversarial Imputation Network

**DOI:** 10.3390/s21072515

**Published:** 2021-04-03

**Authors:** Bubryur Kim, N. Yuvaraj, K. R. Sri Preethaa, Gang Hu, Dong-Eun Lee

**Affiliations:** 1 Department of Architectural Engineering, Dong-A University, Busan 49315, Korea; rlaqjqfuf@dau.ac.kr; 2 Department of ICT Integrated Ocean Smart Cities Engineering, Dong-A University, Busan 49315, Korea; 3 Department of Artificial Intelligence and Data Science, KPR Institute of Engineering and Technology, Coimbatore 641407, India; k.r.sripreethaa@kpriet.ac.in; 4 School of Civil and Environmental Engineering, Harbin Institute of Technology, Shenzhen 518055, China; hugang@hit.edu.cn; 5 School of Architecture, Civil, Environment and Energy Engineering, Kyungpook National University, 80, Daehak-ro, Buk-gu, Daegu 41566, Korea

**Keywords:** wind-pressure coefficients, wind load, machine learning, data imputation, generative adversarial imputation network, tall building

## Abstract

Wind tunnel testing techniques are the main research tools for evaluating the wind loadings of buildings. They are significant in designing structurally safe and comfortable buildings. The wind tunnel pressure measurement technique using pressure sensors is significant for assessing the cladding pressures of buildings. However, some pressure sensors usually fail and cause loss of data, which are difficult to restore. In the literature, numerous techniques are implemented for imputing the single instance data values and data imputation for multiple instantaneous time intervals with accurate predictions needs to be addressed. Thus, the data imputation capacity of machine learning models is used to predict the missing wind pressure data for tall buildings in this study. A generative adversarial imputation network (GAIN) is proposed to predict the pressure coefficients at various instantaneous time intervals on tall buildings. The proposed model is validated by comparing the performance of GAIN with that of the K-nearest neighbor and multiple imputations by chained equation models. The experimental results show that the GAIN model provides the best fit, achieving more accurate predictions with the minimum average variance and minimum average standard deviation. The average mean-squared error for all four sides of the building was the minimum (0.016), and the average R-squared error was the maximum (0.961). The proposed model can ensure the health and prolonged existence of a structure based on wind environment.

## 1. Introduction

With the advancements in construction technologies, numerous large-span roof structures have been built, and clusters of tall buildings are abundant. These flexible, lightweight structures with low damping ratios and low frequencies are very sensitive to wind loads [1]. When wind passes through these tall buildings, wind loads acting on the buildings will reduce or amplify the loads equally [2]. The amplified wind loads may cause severe vibrations in buildings and discomfort to the occupants [3,4]. The building shapes play a vital role in determining wind-flow patterns and wind loads [5,6]. Wind effects on these structures may be low, moderate, strong, or extremely destructive. Moderate and low winds are beneficial, whereas strong and destructive winds may cause structural damages [7]. Additionally, changes in the wind environment around a building may affect the structural durability of nearby buildings [8]. Hence, considerable research efforts have focused on the changes in the wind environment, wind flow, and wind pressure characteristics in tall buildings [9,10,11]. It is thus necessary to understand the characteristics of the wind effects on each structure.

The structural safety of tall buildings depends on their structural designs and their capacities to withstand wind loads and wind-induced vibrations [12]. To understand the wind effects on a structure, it is essential to obtain wind load information for that particular structure [13]. Wind loads can be obtained from a wind tunnel test on a rigid structural model [14]. Wind tunnel tests focus on the measurement of the wind loads on structures, structural responses, and cladding effects on structures under different wind conditions [15]. The wind tunnel test also helps in evaluating the wind pressures generated on the surfaces of buildings with different shapes [16]. Pressure taps are installed on the surfaces of structures to collect wind-pressure values. It is necessary to find an effective method to predict wind pressures on the entire surface of a building even in the absence of, or with faulty pressure taps [17].

Rigid model testing, namely high-frequency base balance (HFBB), provides overall wind loads. The HFBB results can be analyzed using frequency- or time-domain techniques. The HFBB technique is based on concurrent measurements at different locations on the outer surfaces of buildings [18,19]. The wind tunnel test produces wind pressure coefficients, which can be used to analyze the effects of wind loads on buildings. Technological advancements render it promising for instantaneous measurement of wind pressures at more than 1000 locations on a building model with the deployment of pressure taps [20]. The autonomous monitoring of tall buildings was increased following the installation of various types of sensors to assess structural deformation [21], model parameters, and major stress on buildings [22,23]. Long-term building monitoring and maintenance is a necessary task that increases the longevity of buildings [24,25,26,27,28]. However, the functionality of the sensors diminishes with the effects of time and external factors, such as intense pressure and microparticles, which can damage the electronic circuits.

**Figure 1:**
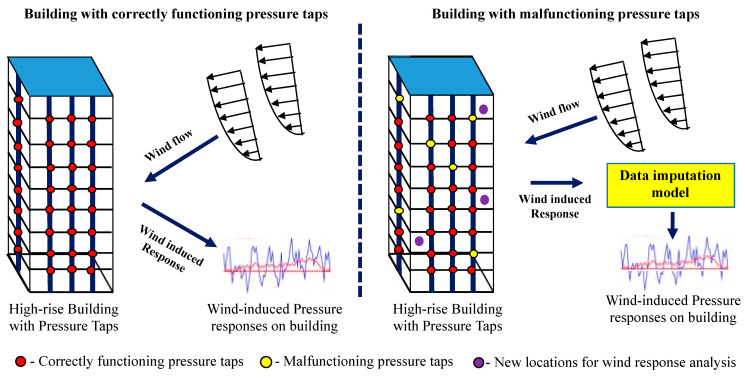
Data imputation model for analyzing wind-induced response.

In addition, the pressure sensors installed in a building for monitoring wind pressure may fail in due course owing to vibration, shock, variation in pressure, electrical interference, and chemical damage. In tall buildings, it is very difficult to identify and replace faulty pressure taps at different locations. In such cases, data relayed from pressure sensors may be corrupted or missing. Data-centric research based on these data face a tremendous threat due to these inevitable data losses. Figure 1 illustrates the need for a data imputation model to analyze the wind-induced pressure response for the buildings with malfunctioning pressure taps and wind response at the new locations of the building without pressure taps installed. Thus, numerous techniques have been developed for imputing missing pressure tap values that may arise owing to tap failure or data loss [29,30]. Missing values are imputed by analyzing the correlation between other pressure coefficients [31]. Optimization techniques estimate the randomly missing time series pressure coefficients [32,33], and sampling techniques have been used to reconstruct the lost data [34].

However, most works related to data imputation in the existing literature focus on predicting the missing data from of multiple points at single time instances. Imputing the missing data for multiple points at multiple time instances remains challenging. This study focuses on handling the data loss by predicting the wind-induced pressure coefficients at different time intervals using a generative adversarial iterative network (GAIN). The performances of the models were quantified using standard statistical measures. GAIN is a light-weight ML model, and its performance shows that it predicts the values more accurately than the other deep learning algorithms applied in the literature [35,36,37]. The remainder of this paper is organized as follows. In Section 2, we summarize the related works in data imputation by machine learning and deep learning algorithms. Section 3 describes the process involved in generating the wind pressure data for buildings with a square section. Section 4 presents the proposed GAIN model and its implementation for predicting missing time series values. Section 5 presents the results and a comparative study of the proposed ML models for the prediction of the missing values. Section 6 concludes the paper and discusses the future scope.

## 2. Related Works

In the literature, artificial neural network (ANN)-based multilayer perceptron (MLP) models were used to estimate the pressure coefficients on the walls and roof of a building by considering the building geometry and wind attack angle [38]. ANNs were used to predict the root-mean-square pressure coefficients of the buildings and the wind-induced pressure at different time intervals on various structures [39,40]. MLP and decision trees were implemented to analyze the nonlinear relationship between the various environmental factors for predicting the deformation of the unstable slopes [41]. Convolutional neural network (CNN) is a deep learning approach which is mostly applied for image-related applications. Regarding predictions, CNN is applied to analyze the wind-induced responses of high-rise structures. Time series data in the time and frequency domain were set as input to the CNN model. CNN models were used to predict the strains in the columns of buildings based on future wind loads with measured wind-response data [42]. Back Propagation Neural Network (BPNN) was implemented for time series predictive model to analyze the displacement based on the captured environmental factors [43]. BPNN and fuzzy neural network were developed to predict the wind pressures on a large gymnasium roof at different time intervals [44]. Subsequently, BPNN was integrated with proper orthogonal decomposition (POD-BPNN) to predict the wind pressure coefficients. The results showed that POD-BPNN was effective in predicting the individual pressure data of a tall building with the minimum number of pressure taps [45]. The Autoregressive recurrent networks (ARN) model based on time series was implemented for identifying the slope displacement [46,47]. ARN models find it difficult to operate on the missed data and impute it with meaningful time series values. Deep neural network (DNN) integrated with long short-term memory (LSTM) is used for the regression analysis to analyze the time series data [48,49]. A deep learning-based autoencoder network is implemented for landslide susceptibility prediction [50]. LSTM-based DNN manages to predict the discontinuity in the electrical resistivity, but dependency on the optimization of the large number of the hyperparameters remains as a major limitation during the time series prediction. Missing values can be imputed using any of the deep learning models, such as ANNs, CNNs, and recurrent neural networks [51,52,53]. Although neural-network-based models can predict wind pressures on structures, the prediction of wind pressure data at multiple points of the structures at different time intervals remains challenging [54]. 

Machine learning (ML) models are preferred over deep learning models for the prediction of missing wind pressure values due to pressure tap failure [55,56]. Random forest (RF) is one of the most widely applied ensemble methods to train the data for prediction by aggregating multiple weak learners [57]. The Extreme Gradient Boosting Regressor (XGBoost) algorithm [58] was implemented for forecasting short-term load in the power plant units. XGBoost struggles to attain the maximum performance with the sparse time series data of a large dataset [59,60]. Although both ML and deep learning work well for data imputation, the deep learning model uses multiple parameters for imputation, resulting in data overfitting [61,62].

To avoid overfitting, an ML-based shallow approach is deployed in this study to compute missing values. In addition, the proposed ML model can be trained and implemented with a smaller amount of data [63]. The missing values in several incomplete, multivariate datasets are imputed using many ML models such as singular value decomposition, general iterative principal component imputation, regularized expectation maximization, truncated total least-squares expectation maximization, K-nearest neighbor (KNN), multiple imputations by chained equations (MICE), and generative adversarial iterative network (GAIN) [64,65,66,67]. The KNN algorithm is used for short-term wind speed forecasting [65]. MICE is found to be more flexible in imputing categorical and quantitative (including skewed) variables [64]. GAIN was implemented on five real-world datasets obtained from the University of California (Oakland, CA, USA), Irvine repository to quantitatively evaluate the imputation performance. It was observed that GAIN models significantly outperformed the other state-of-the-art data imputation methods [68,69,70]. A comparison about the models used in the literature for data imputation and prediction is presented in Table 1.

## 3. Materials and Methods

Analyzing the wind-induced response on the high-rise structures remains important for observing the impact of wind on the structures. As the impact of the wind directly affects the durability of the structures, wind-induced responses on the structures plays a vital role to enhance the longevity of the structures with effective structural maintenance [71,72,73,74]. The technical improvements in implementing the pressure sensors around the high-rise structures for monitoring the wind-induced response gained its maximum attention. Furthermore, the advancements in the field of artificial intelligence (AI) leads a way to develop an integrated system to analyze the deep insights out of the data generated from the pressure sensors. The operational failures and malfunctions that occurs in the pressure taps produce the missing data. Analyzing the wind-induced response from the missing data will not lead to a meaningful insight. In addition, it is also necessary to develop a model which is capable of analyzing the wind-induced response of the building at random locations of the high-rise structures in the absence of pressure taps [75,76,77]. This proposed work concentrates on developing a data imputation model capable of imputing the missing values. Section 3.1 discusses the wind-tunnel test setup for generating the wind-induced response data. An overview of developing an intelligent data prediction model is discussed in Section 3.2.

### 3.1. Wind Tunnel Test and Wind Pressure Data 

**Figure 2:**
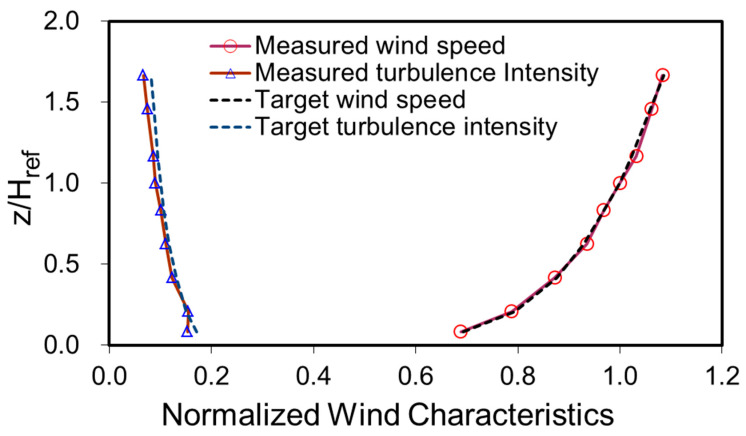
Wind profiles in the wind tunnel. z and H_ref_ represent the heights of the measured position and building top, respectively.

In this study, an aerodynamic database of wind pressures on tall buildings was constructed based on wind tunnel experiments. A tall building model with a square section and dimensions of height (*H*): 600 mm, width (*W*): 120 mm, and breadth (*B*): 120 mm was used in the pressure measurement tests. A synchronous multi-pressure measurement system was employed because of its capability to simultaneously measure multiple-point local pressures on the building model. The oncoming flow conditions were set in terms of Category 2 terrain according to the Australian/New Zealand standard (AS/NZS 1170.2:2011). The normalized wind characteristics between the measured profiles and target profiles are compared in Figure 2.

The mean wind speed at the top of the building model was 13 m/s. Hence, the Reynolds number was 1.03 × 10^5^, which was higher than the minimum Reynolds number requirement as specified in AWES-QAM-1-2001 [78,79]. The sampling frequency was set to 800 Hz, and the measurement duration was 150 s. The local pressure coefficients were calculated using the following Equation:

(1) $$C_{p\_ij}=\frac{p_{ij}\left(t\right)-p_{0}}{\frac{1}{2}\rho V_{H}^{2}}$$

where *i* = 1, 2, …, 10 indicates the pressure tap level, *j* represents the pressure tap number in each level, *p*_0_ is the local static pressure, *ρ* is the air density, and *V_H_* is the velocity at the top of the building model. In this proposed work the incoming wind flow is considered as 0º on the building model placed in the wind tunnel test. As the wind flows at 0º, the front face of the building is perpendicular to the incoming wind.

**Figure 3:**
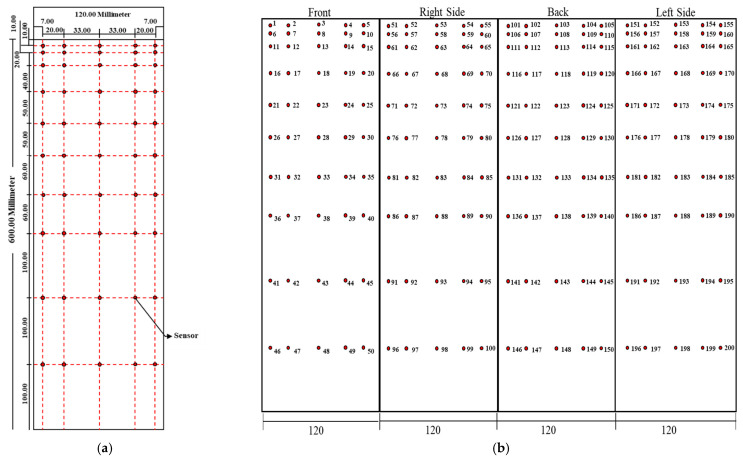
Pressure tap locations (**a**). Relative locations of pressure taps on the proposed building model; (**b**) pressure tap locations on all four sides of the building.

A total of 200 pressure taps were distributed on all four faces of the building, and each face had 50 pressure taps. Figure 3a illustrates the arrangement of pressure taps on the building model, and Figure 3b shows the pressure tap locations on the four faces. The measured wind pressures on the building surface were utilized as a database to train the ML model to predict wind pressures on untested locations of the proposed building.

Data preprocessing yields the most significant outcome of any data-based study. Corrupted data and missing values remain a challenge for data-driven research. Computing a missing value has been a lifesaver for such studies. Conventionally, data imputation is performed with simple mathematical computations, such as imputation using mean/median values, using the most frequent values, or simply using a constant value. However, these conventional imputation techniques have flaws, such as data inaccuracies, data bias, and effects on data correlations. ML-based imputation models, such as GAIN, KNN, and MICE, have been proven to be effective techniques for predicting missing values while maintaining the data accuracy and intactness. In recent years, GAIN has revolutionized many fields through its implementation of the ML paradigm [70]. GAIN has transformed data imputation with its highly accurate imputation predictions.

### 3.2. Intelligent Data Prediction Model

**Figure 4:**
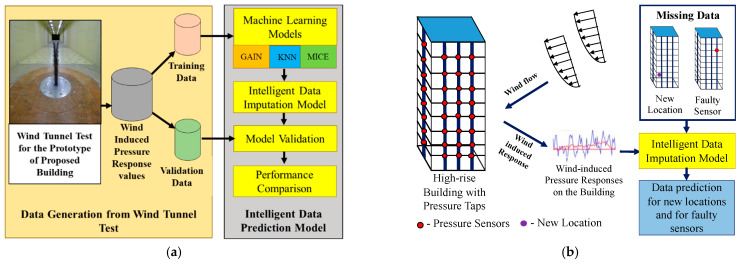
IDPM model. (**a**) Workflow of machine-learning-based intelligent data prediction model for wind pressures; (**b**) workflow of intelligent data prediction model to predict the missing values.

This study aims to build an intelligent data prediction model (IDPM) using ML algorithms. The GAIN-based data prediction model is adopted to impute the missing pressure data at locations with faulty sensors or without pressure taps. The pressure values predicted by GAIN are validated by comparing the model’s performance with those of KNN and MICE. Figure 4a presents the workflow of the ML-based intelligent data prediction model. Eighty-five percent of the measured pressure data were used for training the model and the rest of the data were used for model validation.

Figure 4b presents the workflow of the IDPM. For each face with 50 pressure taps, the data from 1000 instantaneous time intervals data of 50 pressure taps for each side of the building is considered. For each face with 50 pressure taps, wind pressures acquired from all 50 pressure taps were used for training the model among which the values from 7 pressure taps were intentionally imputed with NaN (Not a Number) values to represent the missing values and used for model validation. The NaN was included at two different patterns, namely, missing at random (MAR) and missing completely at random (MCAR). This ensures that 15% of the data contains missing values among the entire data considered for training. Eighty-five percent of the wind pressures measured at 1000 time intervals in the wind tunnel tests were used for training; the rest were used for testing.

## 4. Construction of Wind-Induced Pressure Prediction Model

### 4.1. GAIN

GAIN is a method that generalizes the well-known generative adversarial network (GAN) framework, which can impute data when complete data are unavailable. GAIN is composed of two networks: generator and discriminator. The goal of generators is to impute the missing data, and the discriminator attempts to distinguish the observed components and imputed components [53]. These two networks are trained in an adversarial manner so that the discriminator minimizes the classification loss, and the generator is trained to maximize the classification loss of the discriminator.

In the process of data imputation, the generator (G) imputes the missing components and outputs a completed vector to the discriminator (D). Then, D attempts to determine which components were actually observed and which were imputed. The hint vector (H) is used to provide additional information to ensure that D forces G to learn. H reveals partial missing information to D to focus its attention on imputation quality. In addition, H ensures that G learns to generate the true data distribution.

**Figure 5:**
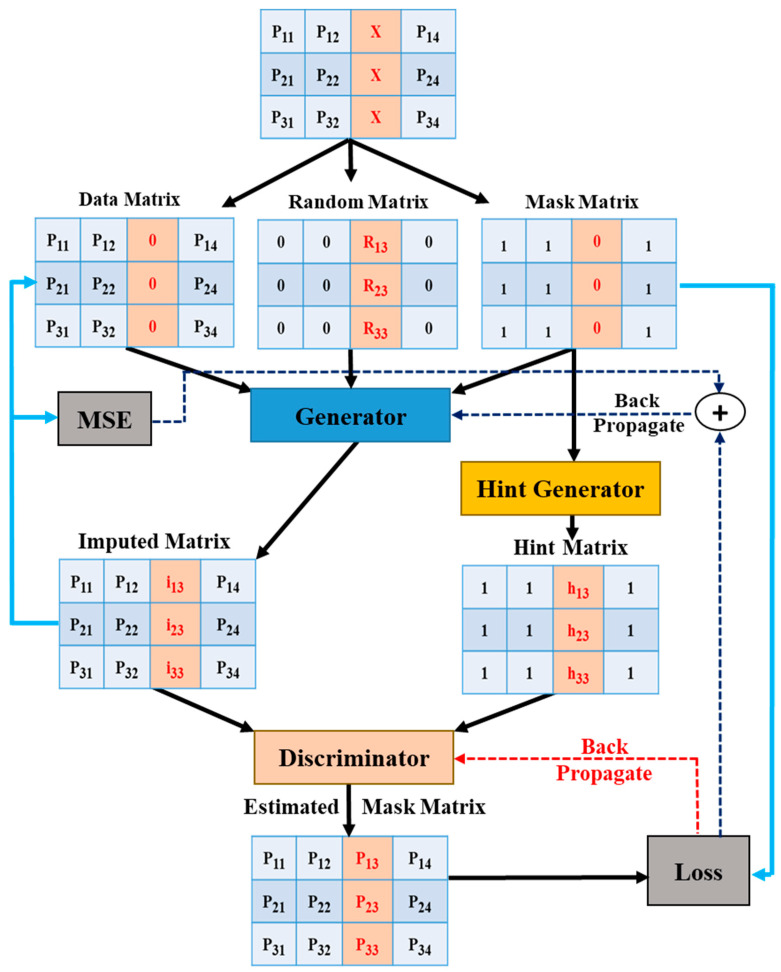
Operation of generative adversarial imputation network (GAIN).

GAIN is the new state-of-the-art algorithm for data imputation, which uses a modified GAN architecture (Figure 5). The pressure coefficients are obtained from 50 pressure sensors in the form of a 10 × 5 matrix. Some specific values in the matrix are converted to NaN values to extrapolate the malfunctioning sensor and missing sensor values. The reshaped matrix of pressure coefficients is transformed into data, random, and mask matrices. The data matrix is an instance of the input that retains all the available sensor values in position but is filled with zeros instead of NaN for missing values. The random matrix consists of randomly generated values in place of the missing values, and the rest of the sensors’ actual values are changed to zeros. The mask matrix is a binary matrix that indicates the missing positions in a matrix using zeros in place of the missing values and ones for occupied positions.

Using these three matrices, G then generates an imputed matrix. This imputed matrix and the data matrix are compared to calculate the mean-squared error (MSE). The hint generator generates a hint matrix depending on the mask matrix, which ensures that D forces G to learn. D outputs the estimated imputed matrix, which is compared with the mask matrix, and the loss is calculated in terms of D’s performance of correctly identifying the missing value. It is used to back propagate to change D’s weights. This loss found in D is also given in summation with the MSE found before G for back propagation. This back propagation is performed until the optimum results are achieved. Both networks are said to learn the parameters during back propagation.

#### Missing Data Imputation Using GAIN

The prediction of the pressure values at the missing locations is formulated as a data imputation problem. Consider a d-dimensional space X = X1 × … × Xd. Represent X = (X1, …, Xd) as a random variable taking values in χ with a distribution P(X).

Let M = (M1, …, Md) be a random variable that takes values as {0,1} in d-dimensional space. X represents the data vector and M is the mask vector. For each i ϵ {1, …, d}, denote $\tilde{\chi_{i}}$ = χi ꓴ {*}, where * is a point that does not belong in any χ_i_, and it represents an unobserved value.

Let $\tilde{\chi }$ = ${\tilde{\chi }}_{1}$ ×…× ${\tilde{\chi }}_{d}$ and $\tilde{X}$ = (${\tilde{X}}_{1}$,…, ${\tilde{X}}_{d}$), where

(2) $$\bar{X}i_{\;}=\;\{\begin{array}{c} X_{i,\;if\;M_{i\;=}1}\\ {*}_{,}\;Otherwise \end{array}$$

M indicates which components of X are observed.

In imputation, the goal is to impute the unobserved values in each $\chi_{i}$. To compute the unobserved values, it is necessary to generate samples according to *P*(X|$\tilde{X}$ = ${\tilde{x}}^{i}$ ). The conditional distribution of X is represented as $\tilde{X}$ = ${\tilde{x}}^{i}$ and is used to fill missing data points. To impute the missing values in the data collected, a vector with real data with some missing values is provided as an input to G, and the missing values are imputed accordingly. D takes the imputed data and determines which data were originally missing.

G takes the value of $\tilde{X}$ as input along with the Z and M values. M is a noise variable, Z denotes the noise vector, and $\tilde{P}$ is the output variable with the missing values being imputed. Let G: $\tilde{p}$i × {0,1}n × [0,1]n → X be a function, and Z = (Z1, …., Zn) be a n dimensional noise variable. The random variables $\tilde{P}$, $\hat{P}$ Ꞓ X are expressed by

(3) $$\bar{P}=G(\tilde{P},M,(1-M)⊙Z)$$

(4) $$\hat{P}=M\;⊙\hat{P}\;+(1-M)⊙\bar{P}$$

where ʘ denotes element-wise multiplication, $\bar{P}$ corresponds to the vector of the imputed values, and $\hat{P}$ corresponds to the completed data vector. This is similar to the standard GAN with Z being analogous to the noise variables in the framework. D is introduced and used as an adversary to train G. In a standard GAN framework, the output of the generator is either completely real or completely fake. To identify the entire vector as real or fake, the discriminator attempts to distinguish the real components and fake components for predicting the mask vector, m. The discriminator function is given as D: *P* → [0,1]^n^ with the i-th component of D ($\hat{P}$) corresponding to the probability that the i-th component of $\hat{X}$ is observed.

A hint mechanism is introduced to avoid the failure of the missing data imputation algorithm. If sufficient information about **M** is not provided to D, then multiple distributions can be generated by G that are optimal with respect to D. To overcome the data insufficiency, the hint-matrix mechanism is followed. H depends on M, and for each imputed sample ($\hat{P}$, m), h is drawn according to the distribution H|M = m. h is passed as an additional input to D, and thus it becomes a function D: P × H→ [0,1]^d^, where the i-th component of D ($\hat{P}$, h) corresponds to the probability that the i-th component of $\hat{P}$ was observed subject to the condition that $\hat{P}$ = $\hat{P}$ and H = h. By defining H in different ways, the amount of information contained in H about M is controlled.

The goal of the discriminator is to maximize the probability of the successful prediction of M, and the goal of the generator is to minimize this probability. Then, define the quantity *V*(D,G) as

(5) $$V(D,G)=\;E\hat{p},M,H\;[M,\log D(\hat{X},H)+(1-M),\log(1-D((\hat{X},H))]$$

where *log* is the element-wise algorithm, and $\hat{X}$ depends on G. The imputation model is associated with the standard GAN, and the objective of GAIN is then defined to minimax the problem as

(6) $$\underset{G}{\min}\;\underset{D}{\max}V\left(D,\;G\right)$$

Define the loss function ℒ: {0,1}^d^ × [0,1]^d →^ Ɍ by,

(7) $$ℒ\left(a,b\right)\;=\;\sum_{i=1}^{d}a_{i\;}\log b_{i\;}+\left(1-\;a_{i}\right)\log(1-b_{i})$$

By expressing $\hat{M\;}$ = D ($\hat{X}$, H), (6) can be rewritten as,

(8) $$\underset{G}{\min}\;\underset{D}{\max}E\left[ℒ\left(M,\;\hat{M}\right)\right]$$

The working code explaining the functionality of GAIN (Algorithms 1) for the prediction of the wind-induced pressure values is presented in the following section.
**Algorithms 1** GAIN for data imputation.1. **While** training loss has not converged **do** 2. **Discriminator (D)** 3. Get $k_{D}$ samples from the dataset ${\left\{\left(\bar{x}\left(j\right),\;m\left(j\right)\right)\right\}}_{j=1}^{k_{D}\;}$ 4. Get $k_{D}$ independent and identically distributed samples ${\left\{z\left(j\right)\right\}}_{j=1}^{k_{D}}$ of Z 5. Get $k_{D}$ independent and identically distributed samples ${\left\{b\left(j\right)\right\}}_{j=1}^{k_{D}}$ of B 6. **For** j = 1… $k_{D}$
**do** 7. $\bar{x}\left(j\right)\leftarrow G\left(\tilde{x}\left(j\right),\;m\left(j\right),\;\;z\left(j\right)\right)$ 8. $\hat{x}\left(j\right)\;\leftarrow m\left(j\right)⊙\tilde{x}\left(j\right)+\left(1-m\left(j\right)\right)⊙\bar{x}\left(j\right)$ 9. h(j)=b(j)⊙m(j)+0.5(1−b(j)) 10. **End for** 11. Update D using adaptive moment estimation optimization (Adam) using the loss obtained from the loss function of D 12. $\nabla_{D}-\;\sum_{j=1}^{k_{D}}{ℒ}_{D}\left(m\left(j\right),\;D\left(\hat{x}\left(j\right),\;\;h\left(j\right)\right),\;b\left(j\right)\right)$ 13. **Generator (G)** 14. Draw $k_{G}$ samples from the dataset ${\left\{\left(\bar{x}\left(j\right),\;m\left(j\right)\right)\right\}}_{j=1}^{k_{G}\;}$ 15. Draw $k_{G}$ independent and identically distributed samples ${\left\{z\left(j\right)\right\}}_{j=1}^{k_{G}}$ of Z 16. Draw $k_{G}$ independent and identically distributed samples ${\left\{b\left(j\right)\right\}}_{j=1}^{k_{G}}$ of B 17. **For** j = 1… $k_{G}$
**do** 18. h(j)=b(j)⊙m(j)+0.5 (1−b(j)) 19. **End for** 20. Update G using Adam (for fixed D) based on the loss obtained from the loss function of G 21. $\nabla_{G}-\;\sum_{j=1}^{k_{G}}{ℒ}_{G}\left(m\left(j\right),\;\hat{m}\left(j\right),\;b\left(j\right)\right)+\;\alpha {ℒ}_{M}\left(x\left(j\right),\;\tilde{x}\left(j\right)\right)$ 22. **End while**

### 4.2. MICE

MICE is one of the principal methods used in statistics to handle missing data. Given that MICE operates by conducting multiple imputations, it is also called sequential regression multiple imputation. MICE operates on multiple imputations to handle the statistical uncertainty caused by the single imputation procedure [54,56]. The chained equation approach in MICE can handle variables of varying types and complexities. Handling missing data by single imputation, such as mean imputation, may result in biased estimation. This can be addressed by multiple imputations. This process involves the filling of the missing values iteratively and the generation of multiple complete datasets.

In MICE, each missing value was predicted multiple times by multiple imputations. The analyses of imputation values were considered to handle the uncertainty and achieve the minimum standard errors. If there is no meaningful information regarding the missing values, the imputation will vary and will lead to high standard errors. In contrast, if the observed data are meaningful and highly predictable, the imputations will be more consistent with more accurate values. MICE can operate based on the assumption that the given variables used in the imputation procedure are missing-at-random and missing-at-complete. The probability of the missing value depends only on the observed values and not on the missing values. The MICE method runs a series of regression procedures, wherein each missing variable is modeled conditionally on other data variables. The regression procedure normally uses logistic regression for binary variables and linear regression for continuous variables.

**Figure 6:**
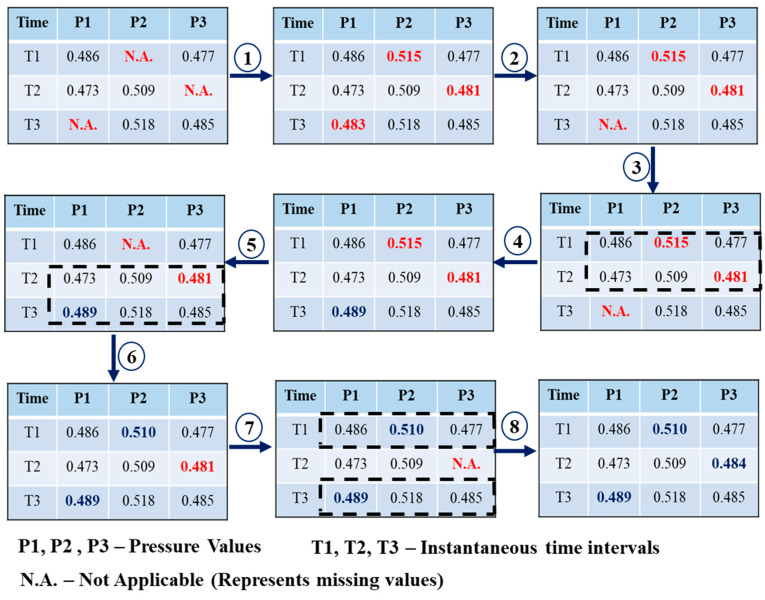
Workflow of multiple imputation by chained equations (MICE).

The MICE algorithm, which operates on the pressure coefficients at various time intervals, is shown in Figure 6.

The chained equation process is explained based on the MICE algorithm for a set of time series pressure values *p_1_*, …., *p_k_*_._ Assuming that some of the pressure values are missing, all missing values are initially filled randomly or with the mean values.

(9) $$\bar{P}\;=\;(\sum^{\;}p_{i})/n$$

where *p_i_* is the time series value and n is the total number of values. This means that the imputation can be considered as the set of filler values. The filler values are obtained by the imputation of one variable and are reset to missing. The first variable with at least one missing value, *p*_1_, is regressed on the other variables *p*_2_, …., *p*_k_. In this regression model, given in Equation (3), variable *p_1_* is a dependent variable, and the other variables act as independent variables. The model operates based on the same assumptions of linear regression while imputing the missing data. The missing value for *p_1_* is predicted by the regression model, and the dataset is imputed with the new predicted values. All missing data are imputed as described above.

*p*_1_ = a + b *p*_2_ + ϵ (10)

where *p*_1_ is the dependent variable, *p*_2_ is the independent variable, a is the intercept, b is the slope, and ϵ is the residual.

Filling each of the missing variables constitutes one iteration. Missing values are predicted at the end of one iteration using the regression relationship observed in the data. The number of iterations is determined based on the error rate calculated at the end of each iteration. The iteration procedure is stopped at a minimum error rate with stable values. The optimum number of iterations depends on the type of data and the number of data values missing in a specific dataset.

### 4.3. KNN

The KNN algorithm is an effective supervised ML algorithm used for data imputation. KNN relies on labeled input data to build a function that produces an appropriate output while handling the missing data. KNN operates based on the assumption that similar data values are in close proximity with each other [57,58]. The KNN measures the similarity between two points by measuring their distance. Although there are different methods to quantify the distance between two points, the most commonly used is the Euclidean distance.

The time series of wind pressures are represented as vectors in a multidimensional feature space. An ML model for data imputation can be developed by using KNN and by considering all the available time series values for training. The value *K* in KNN is a user-defined constant. During the model training process, the KNN algorithm is iterated with multiple *K* values, and the error associated with each *K* value is monitored. This iteration process will be stopped at a particular *K* value when the values are stable and the error rate is minimal. The prediction accuracy of KNN can be improved significantly by selecting the optimum value of *K*.

The KNN algorithm takes wind pressures as input. The dataset is split into a training and testing data. The random number of neighbor values is initialized as *K*. The distance between the pressure points is calculated using the Euclidean measure according to Equation (4).

(11) $$d(x,y)\;=\;\sqrt{\sum_{i=1}^{n}{\left(Y_{i}-{\hat{Y}}_{i}\right)}^{2}}$$

where $Y_{i}$ and ${\hat{Y}}_{i}$ are Euclidean vectors that start from the origin of the space and have specific ending points. *K* nearest points are selected based on the minimum distance measure, and the mean pressure value for all available *k* points is then calculated. The error rate is calculated for the associated *k* values. The procedure is repeated for the next *k* values, and iterations are performed for different seed values. The iteration procedure is stopped when the error rate is minimized. The MSE is calculated for all associated *k* values according to Equation (5).

(12) $$\mathrm{MSE}\;=\;\frac{1}{n}\sum_{i=1}^{n}{\left(Y_{i}-{\hat{Y}}_{i}\right)}^{2}$$

The optimum *K* value is selected based on the accuracy of data imputing. The KNN model is imputed several times. Different *K* values and the optimum values are selected based on the error rate. The prediction becomes less stable when the *K* value decreases. For example, if the value of *K* is selected to be one, it simply replaces the missing value with the nearest value. This will become an unstable value. As the value of *K* increases, the predictions become more stable owing to averaging done by the majority of the neighboring values, and thus more accurate predictions are likely to be made. However, it is possible to witness an increasing value of errors when the *K* value is pushed too far.

## 5. Performance Discussions

**Figure 7:**
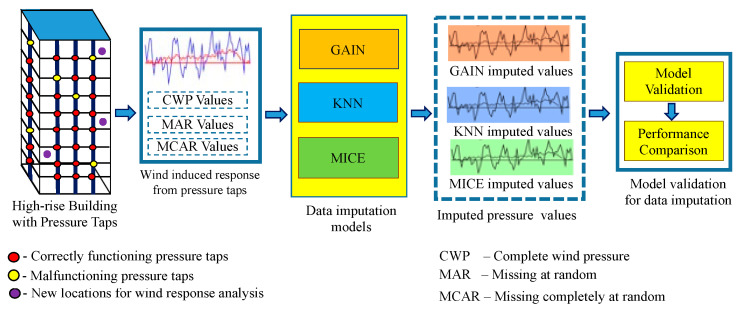
Data imputation models and performance comparison.

This section presents the experimental results of GAIN and the performance comparison of proposed GAIN-based wind-induced pressure prediction with other ML models. In this proposed work, the GAIN model is proposed as an imputation model for filling the missing values generated from the pressure sensors. The performance of the GAIN model is validated by comparing its performance with other familiar ML models, such as MICE and KNN (Figure 7).

### 5.1. Experimental Results of GAIN

**Figure 8:**
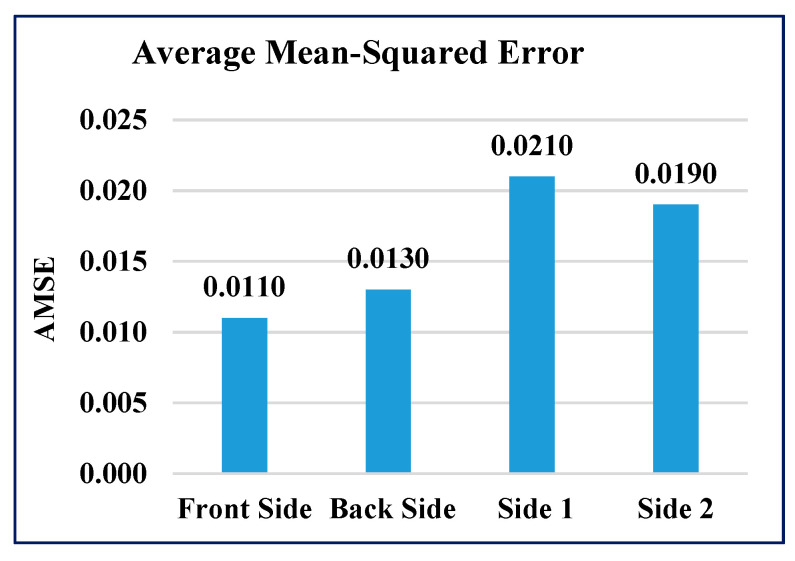
Average mean-squared error (AMSE) of GAIN.

**Figure 9:**
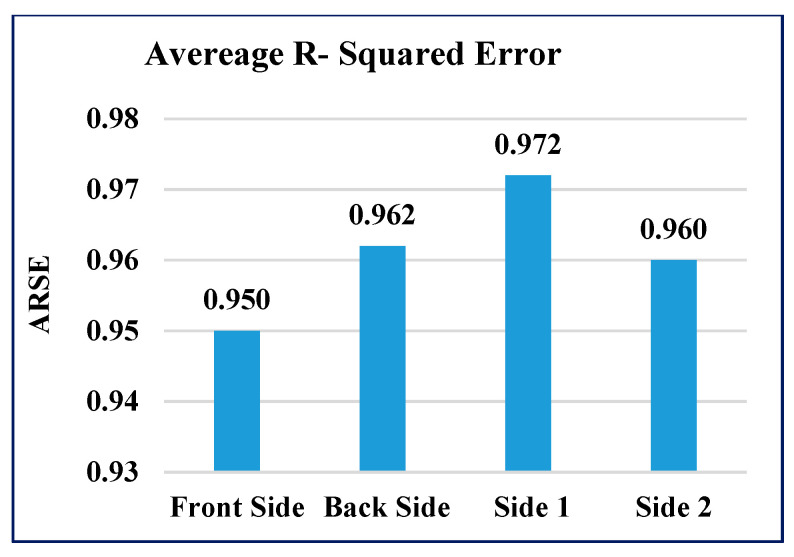
Average R-squared error (ARSE) of GAIN.

The performance of the GAIN algorithm is quantified by calculating the average mean-squared error (AMSE) and average R-squared error (ARSE) values for all four sides of the building. The AMSE of the building was measured to be 0.011, 0.013, 0.021, and 0.019 for front, back, side 1, and side 2 of the building, respectively, as shown in Figure 8. The ARSE values were 0.95, 0.962, 0.972, and 0.96, for front, back, side 1, and side 2 of the building, respectively, as shown in Figure 9.

**Figure 10:**
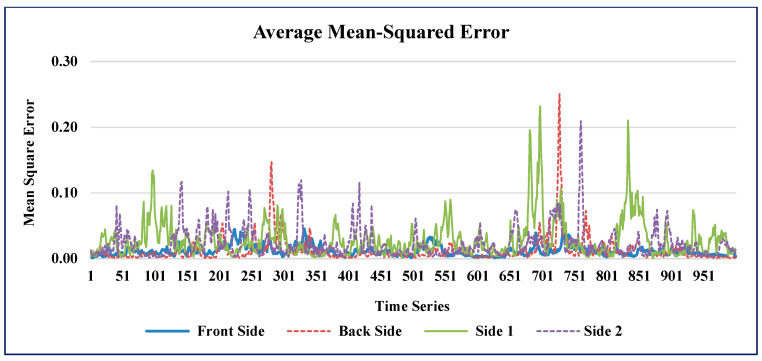
AMSE predicted by GAIN.

**Figure 11:**
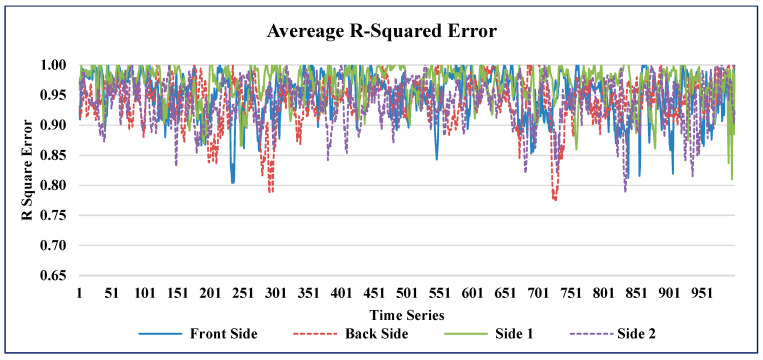
ARSE predicted by GAIN.

The time series plots of the AMSE and ARSE values of GAIN for all four sides of the building are shown in Figure 10 and Figure 11 respectively.

**Figure 12:**
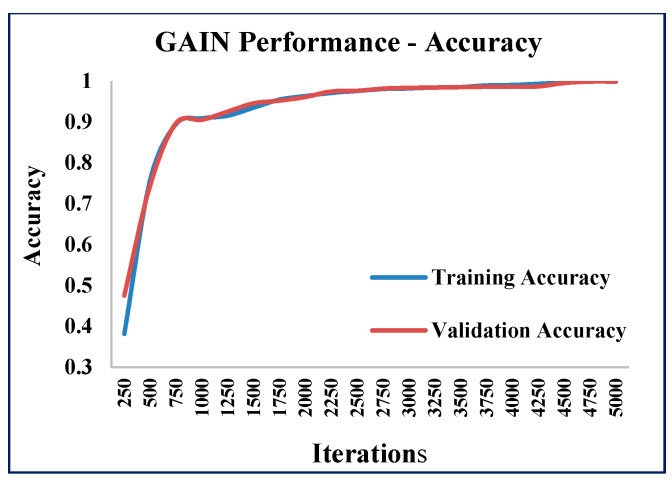
GAIN accuracy measure.

**Figure 13:**
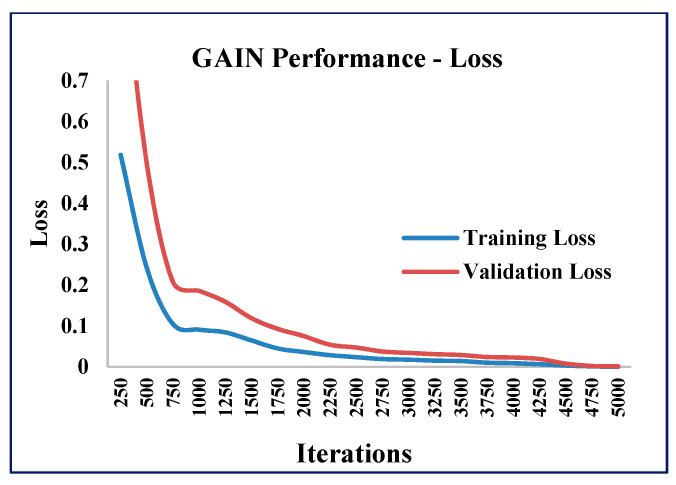
GAIN loss measure.

**Figure 14:**
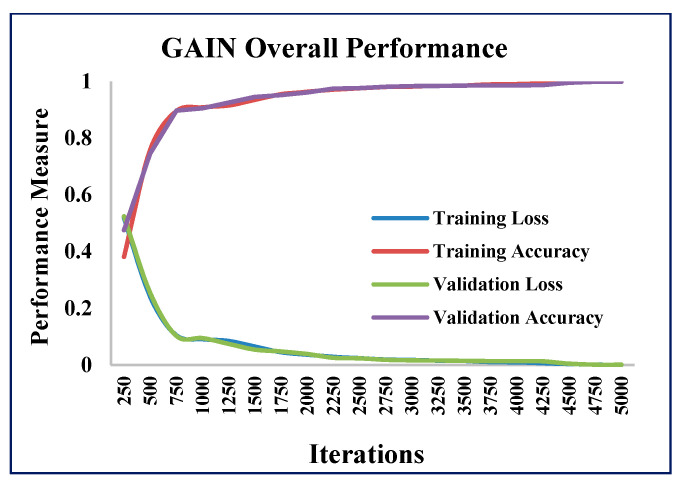
GAIN overall performance.

In addition to the AMSE and ARSE values, the performance of the GAIN algorithm was quantified in terms of accuracy and loss values at different iterations of GAIN. The GAIN algorithm was executed with 5000 iterations and the accuracy and loss values were calculated (Figure 12, Figure 13 and Figure 14).

As the iterations increased, the validation accuracy also increased, and the validation loss decreased. The training and validation accuracies reached the maximum (Figure 12) and stabilized as the iterations increased. The training and validation losses reached the minimum (Figure 13) as the iterations increased.

### 5.2. Experimental Comparisons

The computational performance of GAIN was compared with those of the MICE and KNN models. Initially, the performances of the proposed algorithms were evaluated in terms of AMSE and ARSE by comparing the ground truth and predicted values. During experimentation, MICE was iterated with various *i* values, such as 3, 5, 7, 8, 9, and 10, to identify the best fit for missing values. Among all the iterations, the results at the eighth iteration (*i* = 8) produced the best fit with the AMSE and ARSE values. Similarly, KNN was experimented for different k values ranging from 2 to 9. The results at k = 3 produced the best fit with the minimum AMSE and maximum ARSE values.

**Figure 15:**
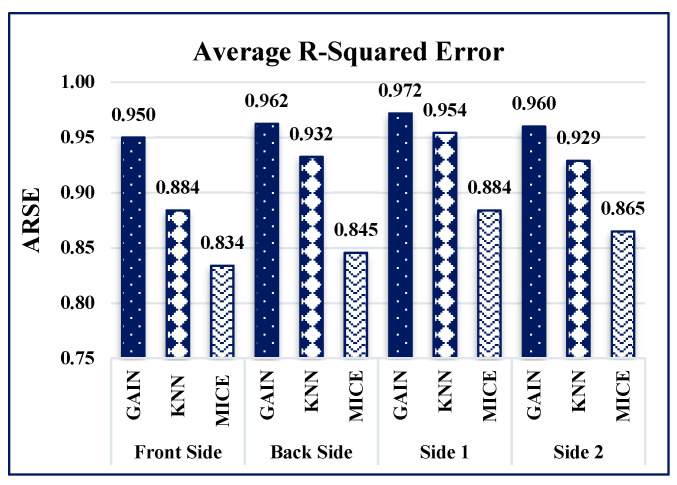
ARSE comparison.

Considering the accuracy of the proposed models, the maximum accuracy in this study was 0.95, and it was achieved by the GAIN prediction model. The MICE and KNN models struggle to attain the maximum accuracy using an iteration model separately for distinct iteration in MICE and by using different k values in KNN. The GAIN model was iterated and the discriminator provided better accuracy with increasing iterations. In addition, the maximum accuracy and minimum loss values were achieved by the GAIN model in successive iterations. However, other models exhibited variations and decreased accuracy, although the k and i values increased. In summary, it is found that the GAIN model works well for finding missing values with high ARSE, low AMSE, decreased loss, and increased accuracy values. GAIN maintains the highest ARSE values (Figure 15) of 0.95, 0.962, 0.972, and 0.962 for the front, back, side 1, and side 2 of the building, respectively. In this proposed work, the building perpendicular to the wind flow is considered for developing the intelligent data imputation model. So, the wind induced responses generated by the pressure taps in the front face of the building is dispersed compared with the other faces of the building. Hence the variance and standard deviation values at the side of the building differs from the sides of the building. 

**Figure 16:**
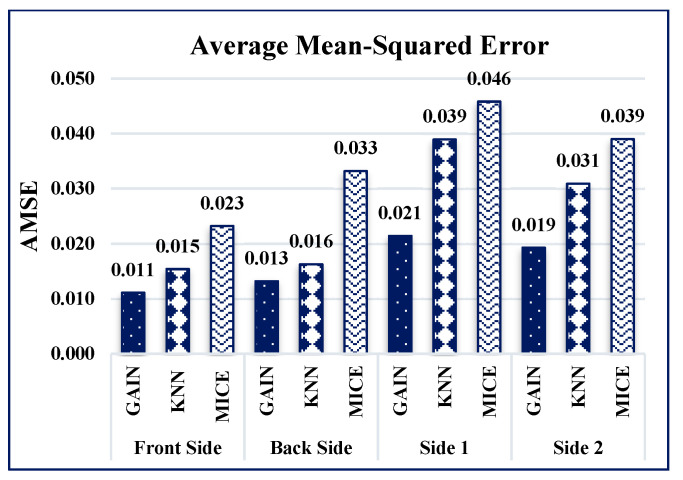
AMSE comparison.

**Figure 17:**
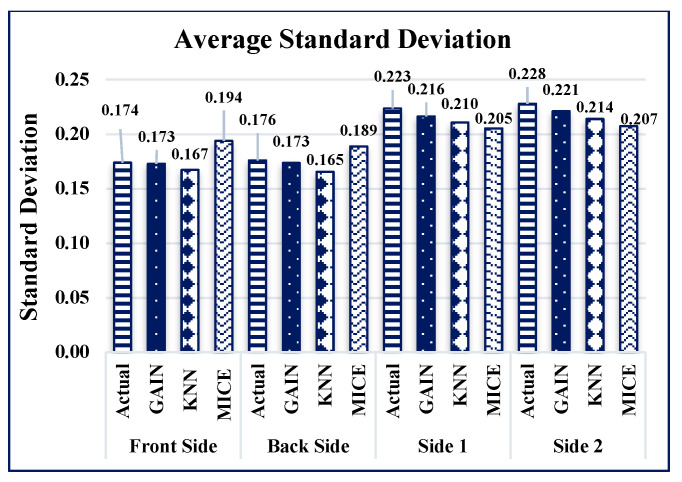
Average standard deviation comparison.

**Figure 18:**
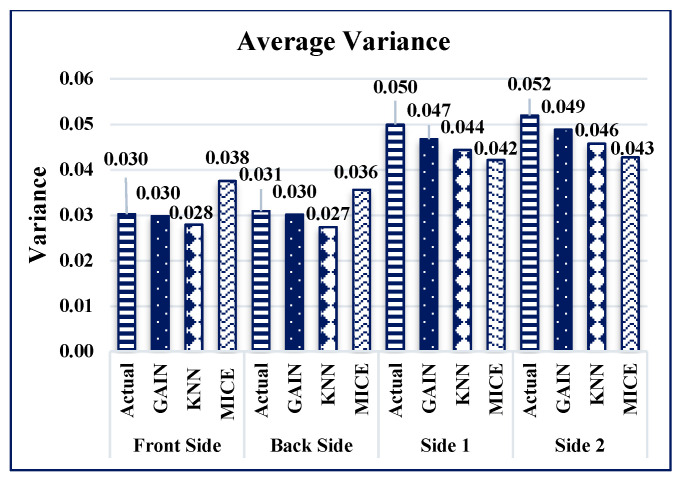
Average variance comparison.

GAIN produces the minimum AMSE values (Figure 16) of approximately 0.011, 0013, 0.021, and 0.019 for front, back, side 1, and side 2, respectively. The KNN algorithm maintains an average AMSE of approximately 0.015, 006, 0.039, and 0.031 for front, back, side 1, and side 2, respectively. The MICE algorithm values deviate considerably from the original values based on the maximum AMSE values in comparison with GAIN and KNN. It is observed that the variance and standard deviation values at the sides of the building remains much closer to each other (Figure 17 and Figure 18). In addition, GAIN manages to produce the minimum standard deviation (Figure 17) and minimum variance (Figure 18). These analyses indicate that the GAIN model is well suited to the prediction of missing pressure value.

**Figure 19:**
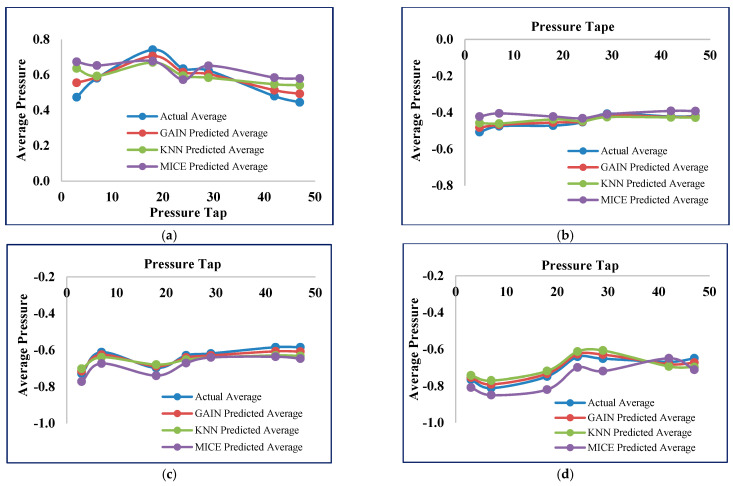
Average pressure value comparison on all sides. (**a**) Average pressure—front side; (**b**) Average pressure—back side; (**c**) Average pressure—side 1; (**d**) Average pressure—side 2.

The comparison of the original pressure values and the imputed pressure values of the MICE, KNN, and GAIN models at different pressure tap locations for all four sides of the building is depicted in Figure 19. It is clearly shown that the GAIN predicted average produces closer imputed values with the actual values when compared with MICE and KNN.

**Figure 20:**
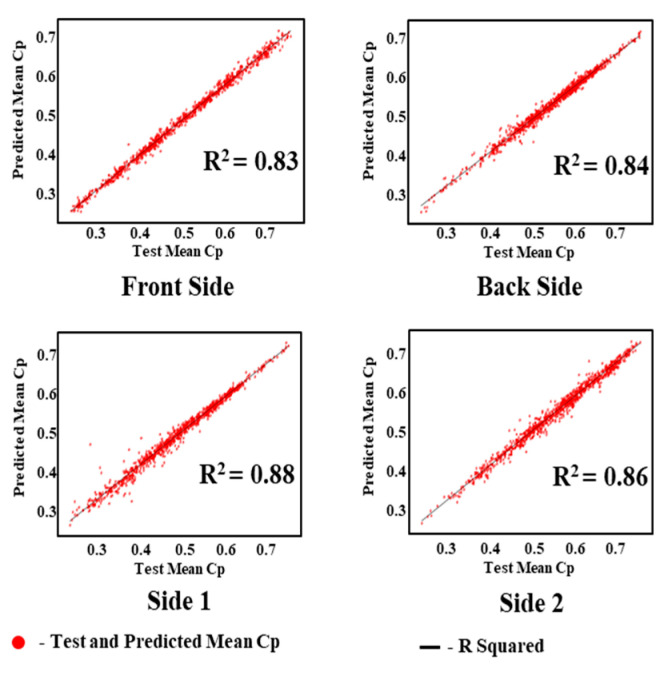
Predicted mean pressure coefficient by MICE.

**Figure 21:**
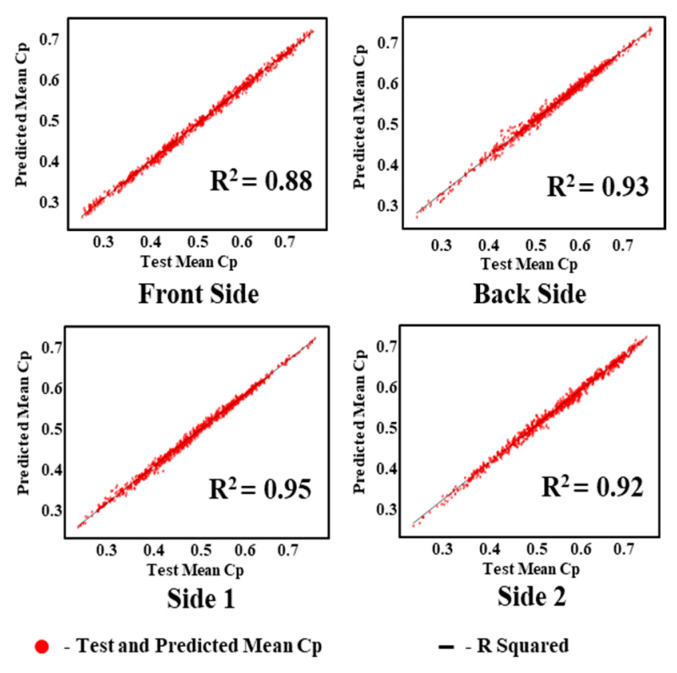
Predicted mean pressure coefficient by KNN.

**Figure 22:**
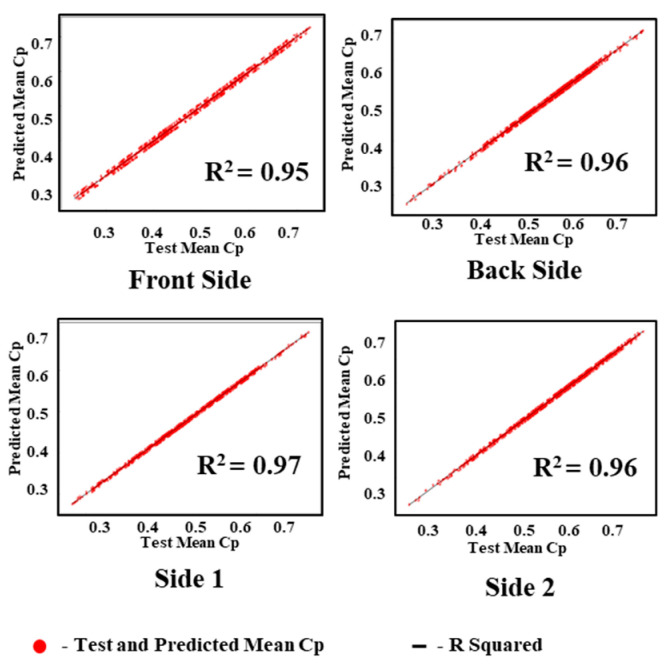
Predicted mean pressure coefficient by GAIN.

The regression analysis values of the test and predicted mean *C_P_* values of KNN, MICE, and GAIN are shown in Figure 20, Figure 21 and Figure 22. It is observed that the test and predicted mean *C_P_* values exhibit the best fit for the GAIN model. The MICE model performs overfitting, and the predicted mean *C_P_* values are scattered. The KNN algorithm can manage the predictions with minimum deviations. The GAIN performance clearly shows that the test and predicted mean *C_P_* values are close to each other with a minimal deviation in comparison with the KNN and MICE algorithms. The performance analyses depicts that the GAIN algorithm can impute the pressure data more accurately than the other ML models.

## 6. Conclusions

Predicting the wind pressure coefficient values at the missing locations plays a vital role in the continuous monitoring of the wind environment around structures. Inevitable data losses and corruption in structural monitoring sensors have tremendous effects on structural health research experiments. In this study, missing wind-induced pressure data at various time intervals of a square-shaped building were predicted by constructing an intelligent data prediction model (IDPM) with ML algorithms. This proposed IDPM model could predict the missing values that occurred owing to faulty sensors. In addition, the IDPM model predicted the wind pressure coefficients on a building by considering the nearby pressure tap values without using multiple pressure taps. A prototype of a proposed building was constructed to conduct the wind tunnel test for data generation. The ML algorithms, namely KNN, MICE, and GAIN, were implemented and validated for predicting missing values. Performance analysis of the proposed ML models indicated that GAIN was more accurate in imputing the missing pressure coefficient values compared with KNN and MICE. GAIN provided a minimum AMSE of 0.01 and a maximum ARSE of 0.96 in comparison with other models. In addition, GAIN exhibited excellent performance with minimum standard deviation and minimum variance between actual and imputed pressure coefficients.

The proposed work applied the best ML algorithms to predict the missing wind pressure values. This study provides a pathway to predict the wind-induced pressure coefficient values of faulty and malfunctioning pressure taps. In addition, it is viable and can predict the wind response at a particular locations on the structure even in the absence of pressure taps. Therefore, the future scope of this work is to minimize the usage of pressure sensors on structures for continuous monitoring of the wind environment.

## Figures and Tables

**Table 1:** Comparison of techniques used for data imputation and prediction.

Technique	Learning Scenarios	Functionality	Pros	Cons
ANN/MLP	Supervised, unsupervised, reinforcement	Modeling data with simple correlations	Naïve structure, easy to build	Slow convergence rate, high complexity, and not suitable for heavy applications
BPNN	Supervised, unsupervised	Modeling the learning derivatives	Fast and simple, efficient for a clean dataset	Sensitive to noisy data, difficult to fix the learning rate
CNN	Supervised, unsupervised, reinforcement	Spatial data modeling	Weight sharing, customizable layer stack arrangement	High computational cost, difficult to optimize the hyperparameters
RCNN	Supervised, unsupervised, reinforcement	Sequential data modeling	Good in capturing the temporal dependencies	Heavily complex model, stuck with vanishing gradient, exploding problems occurs on complex data
ARN	Supervised, unsupervised	Modeling time series and interpretable model	Operates on variety of data and various conditions	Generating variable length output is difficult
Autoencoder	Unsupervised	Dimensionality reduction, compression	Very effective in computation, powerful for unsupervised learning	Pretraining is expensive Stuck with performance for timeseries data
DNN–LSTM	Supervised, unsupervised, reinforcement	Control problems with high dimensional inputs	Fully connected layer arrangement, can overcome vanishing gradient problem.	Depends on large amount of data, very expensive in computation
XG-Boost	Supervised, unsupervised	Modelling less feature engineering applications	Fast in operations, less overfitting	Difficult to optimize the hyperparameters
Randomforest	Supervised, unsupervised	Modelling applications for feature selection	Very effective in highly correlated features	Depend on highly correlated features
KNN	Supervised, unsupervised	Modelling instance-based applications	Easy implementation, evolving model for new data points	Depends on homogeneous features
MICE	Supervised, unsupervised	Data imputations	Flexible, can handle variables of varying types	Sensitive to outliers, depends on homogeneous features
GAIN	Supervised, unsupervised	Data generations	Effective in generating the similar patterns	Convergence is difficult

## Data Availability

Not Applicable.
